# Supplementary material for: Chromothripsis during telomere crisis is independent of NHEJ, and consistent with a replicative origin
Source: Genome Res. 2019 May;29(5):737–49. doi: 10.1101/gr.240705.118 (PMC6499312; doi:10.1101/gr.240705.118)
Supplement: Supplemental Material [file supp_gr.240705.118_Supplemental_file_1.zip › contigs/annotated_contigs/DB89/contig.2.DB89_length_323_mean_cov_14.3839009288.docx]

**DB89_length_323_mean_cov_14.3839009288**

```
TCCCAAACTATTGTATTCATTAAGAGATTCCAATATTTCAGTTTCTCCTCACTTTTCTTCTTTTAGTTTAGTTGTTTATGGAACTTTTC
 >chr4:126710084-126710233 - E=2e-78 p=5e-02
ATTTATCTTTTTTTCTTTCATATTATTTTAAATCTTTCTGTCTTCTAGCCTGTTTACG|GT|TCAGCAGGCCTAAAGGACAGTTCATGG
                                                           >chr4:126708977-126709153 +
AAATTTCTCTGGAAAATAAATCAGAGACCCATAGGAAGGAACCACTAGAGGTCATTCAATCTAACTTTTTGGATATCCCTATAAATATA
E=1e-94
CTTAGATTATGCCCACTTTGAAGCACATATTGTCTAATGTAGTTTATAAAGATATTAA
```
